# Therapeutic Effect of Saponin Rich Fraction of *Achyranthes aspera* Linn. on Adjuvant-Induced Arthritis in Sprague-Dawley Rats

**DOI:** 10.1155/2015/943645

**Published:** 2015-07-26

**Authors:** Pankaj S. Kothavade, Vipin D. Bulani, Dnyaneshwar M. Nagmoti, Padmini S. Deshpande, Nitin B. Gawali, Archana R. Juvekar

**Affiliations:** Department of Pharmaceutical Science and Technology, Institute of Chemical Technology, N. P. Marg, Matunga, Mumbai, Maharashtra 400 019, India

## Abstract

*Objective. Achyranthes aspera* Linn. (AA) is used in folklore for the treatment of various inflammatory ailments and arthritis like conditions. Anti-inflammatory activity of saponin rich (SR) fraction of AA has been previously reported. The objective of this study was to assess the antiarthritic effect of SR fraction of *Achyranthes aspera* in adjuvant-induced arthritic rats. *Methods.* Arthritis was assessed by arthritis score, paw volume, changes in tibiotarsal joint thickness, hyperalgesic parameters, and spleen and thymus index. Haematological, serum, biochemical, and inflammatory cytokine and *in vivo* antioxidant parameters were measured on the last day of the study. *Results.* SR fraction significantly suppressed paw swelling and arthritic score and improved the pain threshold in motility and stair climbing tests. There was a reversal in the levels of altered parameters, alanine aminotransferase, aspartate aminotransferase, alkaline phosphatase, and antioxidant parameters like superoxide dismutase, catalase, glutathione, malondialdehyde, and nitric oxide. SR fraction significantly decreased plasma levels of tumor necrosis factor-alpha and interleukin-6. Moreover, histopathology revealed a significant reduction in synovial hyperplasia, inflammatory cell infiltration, and bone destruction in the joints. *Conclusion.* These observations explain the therapeutic benefit of SR fraction of AA in suppressing the progression of adjuvant-induced arthritis in rats.

## 1. Introduction

Rheumatoid arthritis (RA) is a chronic autoimmune disease that comprises a syndrome of pain, stiffness, and symmetrical synovitis of diarthrodial joints lead to slowly progressive disease characterized by a continual breakdown of articular cartilage and changes in the subchondral bone. RA affects about 1% of the world population and more common in women than in men 3 : 1 ratio [1, 2]. Currently, RA is clinically treated mainly by disease-modifying antirheumatic drugs (DMARDs), such as methotrexate and sulfasalazine, and nonsteroidal anti-inflammatory drugs (NSAIDs) including ibuprofen, aceclofenac, and naproxen combined with steroid hormones like cortisone and prednisone [3]. However, these drugs only transiently suppress inflammation and ameliorate symptoms as curable treatment is not available in the market for the consequences associated with arthritis. Hence, exploration of new antirheumatic drugs with high efficacy and less toxicity is needed.

*Achyranthes aspera* (AA) Linn. (Amaranthaceae) is widely distributed throughout Asia, South America, and Africa [4]. It is an annual, perennial, stiff, and erect herb growing up to one meter in height [5].* A. aspera* is traditionally used for treatment of piles, renal dropsy, cough, pneumonia, kidney stone, skin eruptions, snake bite, gonorrhea, dysentery, asthma, hypertension, and diabetes [4, 6]. AA has been reported for its preliminary anti-inflammatory and antiarthritic activity, antinociceptive, antifungal, immunomodulatory antifertility and abortifacient, antiurolithiatic, and anxiolytic activities [6–12].

Our previous research exhibits saponin rich (SR) fraction of AA possessed anti-inflammatory effect through activity-guided fractionation [13]. Triterpenoid saponins from herbs attracted attention due to their structural diversity and biological activities like antitumor, antifungal, antiviral, and anticoronary heart dieseaes [3]. Therefore, our current study was designed to confirm antiarthritic effect of saponin rich fraction of* A. aspera* and to explore its potential mechanism by ajuvant-induced arthritis in rats.

## 2. Material and Methods

### 2.1. Plant Materials

Whole plant of* Achyranthes aspera* was collected during the month of October 2013 from Thane District, Maharashtra, India. The plant material was authenticated by Professor Ganesh Iyer, Ramnarain Ruia College, Matunga (East), Mumbai. A voucher specimen (2012/02) of the plant was deposited at the Herbarium of the Department of Pharmaceutical Science and Technology, Institute of Chemical Technology, Mumbai, India.

### 2.2. Reagents

Fraud's complete adjuvant (FCA) and indomethacin were purchased from Sigma-Aldrich (St Louis, MO, USA). ELISA test kits were purchased from KRISHGEN BioSystems, India. All other chemicals and reagents used for study were of analytical grade procured from Sigma-Aldrich (St Louis, MO, USA).

### 2.3. Fraction Preparation

The dried whole plant (3.5 kg) was extracted with methanol and filtered. The extract (418 g) was dissolved in water and portioned with petroleum ether, chloroform, ethyl acetate, and* n*-butanol, successively. The bioactive petroleum ether fraction was partitioned with methanol. Methanolic fraction was further taken up in water and reextracted with diethyl ether until all chlorophyll pigments were removed. Then aqueous phase was partitioned with* n*-butanol. The* n*-butanol fraction concentrated to dryness and saponin mixture (5.60 g) was obtained.

### 2.4. Animals

Sprague-Dawley adult female rats (180–200 g) were obtained from the Animals house, Haffkine Bio-Pharmaceutical Corporation Ltd., Mumbai. The animals were maintained, under a 12 hr light/dark cycle, at controlled room temperature (25 ± 1°C) and relative humidity (55 ± 5%). The animals were fed with a rodent standard diet with free access water* ad libitum*. All the animals were acclimatized to the laboratory environment for a week before the experiment. The Animal Ethics Committees of the Department of Pharmaceutical Science and Technology, Institute of Chemical Technology, Mumbai, approved all experimental protocols in accordance with Committee for the Purpose of Control and Supervision of Experiments on Animals (CPCSEA) guidelines.

### 2.5. Adjuvant-Induced Arthritis in Rats

SD rats were randomly divided into five groups, namely, control group, FCA control group, positive control group of indomethacin (1 mg/kg), and SR fraction (50 and 100 mg/kg) groups with six rats in each group. Except the control group, the arthritis was induced by a single injection of 0.1 mL of FCA into the left hind paw on day 0. After this primary immunization, treated groups were orally administered with SR fraction (50 and 100 mg/kg), indomethacin (1 mg/kg), while normal control group and FCA control group were given equal volume of normal saline. All groups were orally administered those doses daily after arthritis induction until the end of experiment (day 28).

### 2.6. Measurement of Arthritic Progression

#### 2.6.1. Measurement of Paw Volume and Tibiotarsal Joint Thickness and Arthritis Score

Paw volume and tibiotarsal joint thickness of the injected paw was measured using plethysmometer (Model no. 7140, UGO Basile, Italy) and digital calliper (Mitutoya, Japan) on days 0, 7, 14, 21, and 28 days after vehicle/drug administration, respectively [14]. The severity of arthritis was assessed on a scale of 0–4 with the following criteria: 0 menas no edema or swelling, 1 means slight edema and limited erythema, 2 means slight edema and erythema from the ankle to the tarsal bone, 3 means moderate edema and erythema from the ankle to the tarsal bone, and 4 means edema and erythema from the ankle to the entire leg. The sum of the scores for all 4 limbs was calculated as the arthritic index, with a maximum possible score of 16 per rat [15].

#### 2.6.2. Dorsal Flexion Pain, Motility Test, and Stair Climbing Ability [16]

In dorsal flexion pain (DFP), FCA injected paw was scored based on squeaking and leg withdrawal after the ankle joint of the rat was flexed five times until the toes touched the front of the leg. The DFP was scored from 0 to 2: score 0, if there were no squeaking and leg withdrawal; score 1, if there is either squeaking or leg withdrawal; score 2, if both squeaking and leg withdrawal were present. Motility was observed for the period of 5 min and scored from 0 to 2 points. Score 0: rats avoided touching the feet to ground; score 1: rats walked with difficulty with toes touching the ground; score 2: rats walked easily. In stair climbing ability test, trained rats were allowed to climb on a staircase with steps at 5, 10, and 15 cm having water at second and food on third step. This stair climbing ability was scored from 0 to 3, score 0 if the rats did not climb any step, score 1 if they climbed onto step 1, score 2 if they climbed onto step 2, and score 3 if they climbed onto step 3.

#### 2.6.3. Thymus and Spleen Index

At the day 28 after immunization, the animals were sacrificed and thymus and spleen were promptly removed and weighed. The index of thymus and spleen was expressed as the ratio of thymus and spleen wet weight versus body weight (mg/g), respectively [14].

#### 2.6.4. Haematological Analyses

On day 28 after FCA injection, blood sample of rat was collected in EDTA containing tubes from retroorbital vein puncture. The total and differential leukocyte counts were determined from blood sample.

#### 2.6.5. Biochemical Analysis in Liver

Animals were sacrificed on day 28 after immunization; the liver was promptly removed and stored at −70°C. Portion of livers was homogenized. The homogenate was centrifuged at 6000 x g at 4°C for 20 min and the supernatant was collected. The supernatant was used to measure superoxide dismutase (SOD), catalase (CAT), reduced glutathione (GSH), malondialdehyde (MDA), and nitric oxide (NO) activity [14].

#### 2.6.6. Biochemical Analysis in Serum and Cytokines Levels in Plasma

The levels of alkaline phosphatase (ALP), aspartate transaminase (AST), and alanine transaminase (ALT) in serum were determined by using quantitative colorimetric assay kits (manufacture name), according to the manufacturer instructions. Interleukin-1 (IL-1) and tumor necrosis factor alpha (TNF-*α*) in plasma were determined by using commercially available rat cytokine ELISA kits, according to the manufacturer instructions [14].

#### 2.6.7. Histopathological Evaluation of Knee Joints

On day 28 after FCA injection, all rats were sacrificed after blood collection. Knee joint of injected paw was removed for histopathological examination. The joints were fixed in 10% phosphate buffer formalin, decalcified in 10% EDTA for 30 days at 4°C, and then embedded in paraffin. Sections of 5 *μ*m were stained with hematoxylin and eosin (H&E) and examined under the light microscope.

### 2.7. Statistical Analysis

Data are expressed as mean ± SEM, and statistical comparisons were carried out using one-way analysis of variance (ANOVA) followed by post hoc Dunnett's test for comparison between control and test groups. The Dorsal flexion pain, stair climbing ability test, and motility are expressed as median scores and the Kruskal-Wallis test was used to compare the groups. The minimal level of significance was identified at *P* < 0.05.

## 3. Result

### 3.1. Effect of SR Fraction on Rat Hind Paw Volume, Tibiotarsal Joints Thickness, and Arthritis Score

**Figure 1:**
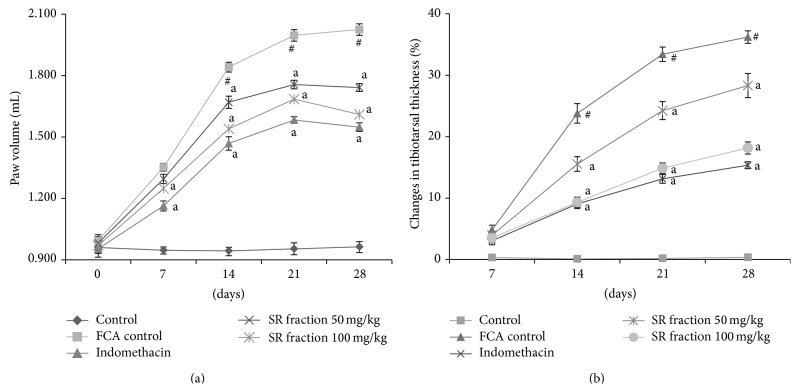
Effect of saponin rich (SR) fraction on (a) paw swelling and (b) % changes in tibiotarsal thickness. All data were expressed as mean ± SEM (*n* = 6). ^a^ *P* < 0.05 compared to the FCA control group. ^#^ *P* < 0.05 compared with vehicle control.

Rat paw swelling trend is shown in Figure 1(a), in which the swelling of the right hind paw increased over time up to day 28. SR fraction (50 and 100 mg/kg) and indomethacin (1 mg/kg) treated group with significantly reduced paw edema compared with FCA control (*P* < 0.05). After day 14, clearly recognizable changes in tibiotarsal joint thickness were observed in the treated groups as compared to FCA untreated group. At day 7, no significant difference was found in tibiotarsal joints thickness of all groups (Figure 1(b)). Arthritis was scored on days 7, 14, 21, and 28 by physical observation. Treatment with SR fraction (100 mg/kg) significantly (*P* < 0.05) suppressed the arthritic score of FCA injected rat hind paw from day 14 to day 28. However, SR fraction 50 mg/kg significantly reduced the arthritic score on day 28 (Table 1).

### 3.2. Effect of SR Fraction on Dorsal Flexion Pain, Motility Test, and Stair Climbing Ability

**Figure 2:**
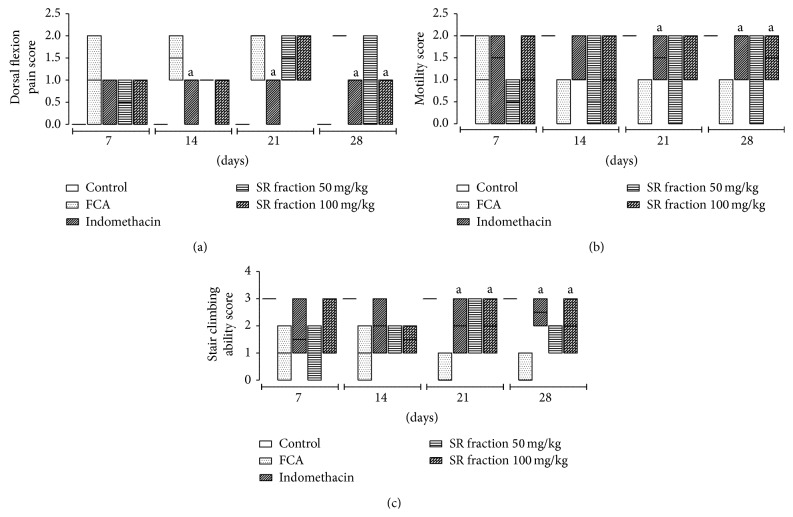
Effect of saponin rich (SR) fraction on inflammatory hyperalgesia in arthritic rats. The scores of (a) dorsal flexion pain, (b) motility, and (c) stair climbing ability is illustrated as box plots where bold line represents median values (*n* = 6) and boxes represent interquartile ranges (25th and 75th percentiles), ^a^ *P* < 0.05 compared with FCA control.

As shown in Figure 2, FCA-induced arthritic rats exhibited low threshold of pain in hyperalgesic parameters such as dorsal flexion pain, motility, and stair climbing ability. In dorsal flexion pain treatment with SR fraction at dose of 100 mg/kg showed significance reduction in median score on day 28 compared to untreated arthritic rats (*P* < 0.05). Treatment indomethacin and SR fraction showed improvement in impaired motility pattern compared to untreated arthritic group. Moreover, SR fraction at dose of 100 mg/kg exhibited a significant (*P* < 0.05) increase in stair climbing score on day 21 and day 28 compared to arthritic control group.

### 3.3. Thymus and Spleen Index

The index of thymus and spleen was assayed on day 28. Results report an increase in thymus and spleen index of FCA control group compared to normal control group. There was significantly (*P* < 0.05) reduction of index of thymus and spleen in SR fraction treatment group (Table 2).

### 3.4. Haematological Examinations

Changes in peripheral blood profile were observed on day 28 after injection of FCA into the hind paw of rats. Indomethacin (1 mg/kg) showed significant decreases in total WBCs count. However, there were no significance differences in the haematological profile of SR fraction treated rats (Table 3).

### 3.5. Biochemical Examination in Liver Homogenate

The effects of SOD, CAT, GSH, MDA, and NO are summarized in Table 4. SOD, CAT, and GSH activities were significantly decreased, whereas MDA and NO level increased significantly in FCA control group compared to normal control (*P* < 0.05). Treatment with SR fraction (50 and 100 mg/kg) and indomethacin (1 mg/kg) showed significant modulation in all above antioxidant indexes. Thus SR fraction has potentially antioxidant effect.

### 3.6. Biochemical Analysis in Serum

Table 5 depicts the effect of SR fraction on levels of tissue marker enzyme in arthritic rat serum. A significant increase in the levels of AST, ALT, and ALP was observed in arthritic rats. The administration of SR fraction (50 and 100 mg/kg) and indomethacin (1 mg/kg) significantly reverted all these indexes compared to FCA control (*P* < 0.05).

### 3.7. Cytokines Levels in Plasma

**Figure 3:**
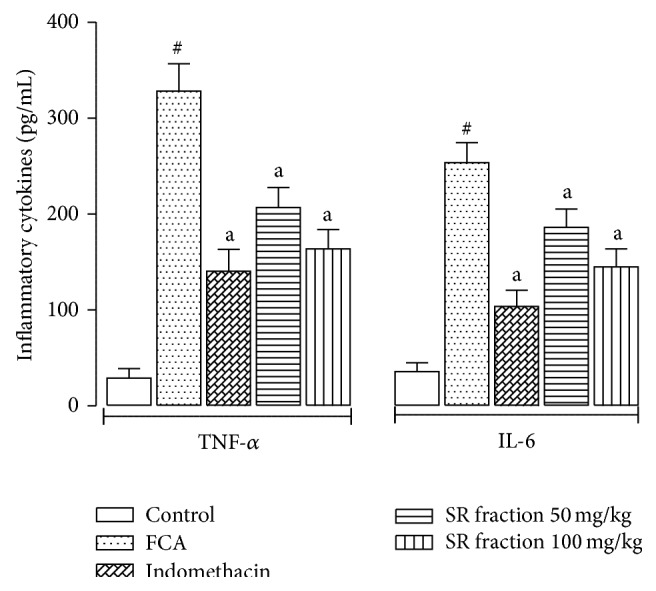
Effect of saponin rich (SR) fraction on inflammatory cytokines, namely, TNF-*α* and IL-6 levels in plasma samples. Values are presented as mean ± SEM, *n* = 6. ^a^ *P* < 0.05 compared with the FCA control group. ^#^ *P* < 0.05 compared with vehicle control.

Plasma levels of inflammatory cytokines TNF-*α* and IL-6 in untreated arthritic rats were significantly higher than those of treatment groups (*P* < 0.05). Both doses of SR fraction 50 and 100 mg/kg markedly inhibited the production of TNF-*α* and IL-6 in arthritic rats (Figure 3).

### 3.8. Histopathological Examination

**Figure 4:**
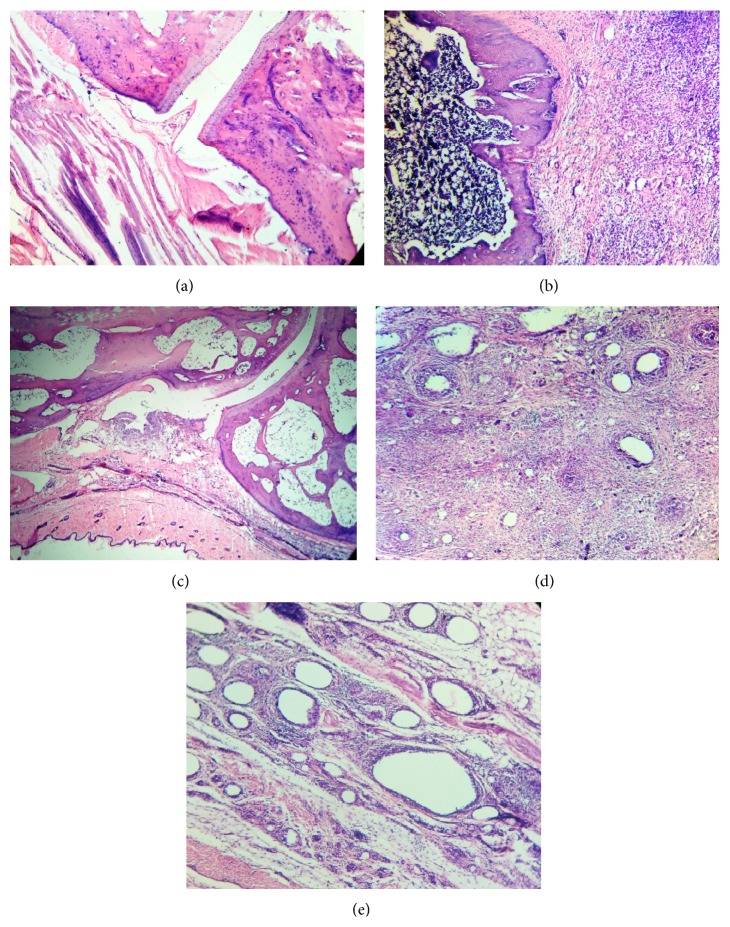
Effect of saponin rich (SR) fraction on histopathological changes of tibiotarsal joints in arthritic rats. All images of one of six in each group. (a) Vehicle control, (b) adjuvant-induced arthritic control, (c) indomethacin (1 mg/kg), (d) saponin rich fraction (50 mg/kg), and (e) saponin rich fraction (100 mg/kg).

Histopathological results are shown in Figure 4. In arthritic group soft tissue swelling around joints were seen with varying degrees of inflammatory cells infiltration, synovial hyperplasia, and cartilage destruction in the joint. Histological images of tibiotarsal joints were improved in arthritic animals treated with SR fraction (50 and 100 mg/kg), as evidence by marked reduction in synovial hyperplasia and inflammatory cell infiltration, which reveals the protective effect of SR fraction in arthritis.

## 4. Discussion

Arthritis is an immune-mediated destruction of joints, characterised by pain, swelling, tenderness, and difficulty in movement. The events that underline the progression of arthritis are mediated by several chemotactic factors such as interleukins (IL-1, IL-6, and IL-10), tumor necrosis factor (TNF-*α*), interferons (INF-*γ*) plus leukocytes, and adhesion factors [17]. The current lines of therapy typically involve the use of immunosuppressants which are accompanied by a range of adverse events, particularly after long-term treatment. The animal model that closely resembles human osteoarthritis is adjuvant-induced arthritis in rats [18]. Subcutaneous administration of killed* Mycobacterium* H37Ra causes activation of immune response, leading to leukocyte accumulation in the joints that subsequently causes pain and inflammation [19]. NSAIDs are effective in alleviating the joint pain and swelling associated with synovial fluid accumulation. However, they are not particularly effective in circumventing the disease progression. Here, we have chosen Indomethacin as the standard treatment because of its clinical effectiveness in human arthritis. In this study, we challenged the rats with 0.1 mL of Freund's complete adjuvant and assessed the progression of arthritis at various stages, in terms of paw volume and tibiotarsal joint thickness. Although significant increase in the paw volume and tibiotarsal thickness was evident only on Day 14, indicating synovial fluid accumulation, the increase was moderately less for indomethacin and SR treated groups (100 mg/kg) on day 14, as compared to negative control group. The effect was observed in the SR (50 mg/kg) group only on day 28.

Pain is an important parameter to assess the effect of joint destruction on motility. Hyperalgesia is important indicator of arthritic development, which can be assessed by the direct measurement of pain and its effects on motility. In this study, SR fraction (100 mg/kg) significantly improved the pain threshold in hyperalgesic rats on day 28. The effect of SR (100 mg/kg) on stair climbing was more pronounced from day 21 onwards, whereas significant increase in motility score was evident only on day 28. Indomethacin consistently showed improved motility and stair climbing score from day 14 onwards; however effects on pain score were evident only after day 20. This suggests that the anti-inflammatory action was more pronounced than effects on pain, which might be attributed to alleviation of proinflammatory cytokines. The findings are further supported by the significant inhibitory effect of SR fractions (50 mg/kg and 100 mg/kg) on the levels of TNF-*α* and IL-6 in the plasma of FCA-challenged rats.

Although the immune factors are responsible for triggering the pathogenic events that lead to arthritis, oxidative events do play a role in the disease progression. According to Halliwel, lipid peroxidation, caused as a result of immunological response, eventually causes increased oxidation that further worsens the condition [20]. The increased oxidative stress produces a decrease in the levels of the inherent antioxidant enzymes glutathione peroxidise, catalase, and superoxide dismutase [21]. With a decrease in antioxidant defence, there is a concurrent increase in the levels of reactive oxygen species and nitric oxide (NO), as a consequence of increased lipid peroxidation indicated by increased MDA [22]. Treatment with SR fraction significantly restored the antioxidant status by decreasing NO and MDA levels and elevating the levels of SOD, catalase, and GSH enzymes.

Clinically, arthritis has been known to result in increased levels of WBCs particularly leucocytes accompanied by enlargement in spleen [23]. The spleen has been reported to be involved in the storage and recruitment of leucocytes across the site of inflammation. Administration of FCA led to increase in the total blood leucocyte count suggesting the involvement of WBCs in response to antigen-mediated arthritic insult. These changes were significantly reversed by SR treated fractions (50 and 100 mg/kg). Treatment with SR fraction (50 and 100 mg/kg) also resulted in significant decrease in the weight of the spleen.

Histopathological analysis of FCA induced arthritic rats showed a mild degree of infiltration of inflammatory cells, synovial hyperplasia, and cartilage destruction in the joint. Treatment with SR (50 and 100 mg/kg) significantly improved the histology of tibiotarsal joint, resulting in decreased evidence of destruction and inflammation.

The results of the present study reveal a significant potential of SR fraction of* Achyranthes aspera* in slowing down the arthritic progression and reversing the pathological changes resulting from arthritic development. This effect can be attributed to a decrease in the level of cytokines, TNF-*α*, and other proinflammatory factors. At the same time, it may be suggested that improvement in oxidative stress may be as a result of direct antioxidant effect of SR fraction coupled with an improvement in the body's natural antioxidant enzyme mediated defence.

## Figures and Tables

**Table 1:** Effect of saponin rich (SR) fraction of *Achyranthes aspera* on arthritis score in arthritic rats.

Treatment	Dose (mg/kg)	Arthritic score ± SEM			
		Day 7	Day 14	Day 21	Day 28
Control	—	0.00	0.00	0.00	0.00

FCA control	—	6.17 ± 0.477	11.50 ± 0.563	13.33 ± 0.494	13.50 ± 0.619

Indomethacin	1	5.67 ± 0.333	8.67 ± 0.333^*∗∗∗*^	9.17 ± 0.477^*∗∗∗*^	7.83 ± 0.307^*∗∗∗*^

SR fraction	50	6.17 ± 0.307	10.83 ± 0.654	11.83 ± 0.477	10.83 ± 0.477^*∗∗*^
	100	5.83 ± 0.307	9.33 ± 0.333^*∗*^	8.50 ± 0.428^*∗∗∗*^	8.00 ± 0.365^*∗∗∗*^

Values are expressed as means ± SEM, *n* = 6.

^*∗*^ *P* < 0.05, ^*∗∗*^ *P* < 0.01, and ^*∗∗∗*^ *P* < 0.001 compared to the FCA control group.

**Table 2:** Effect of saponin rich (SR) fraction of *Achyranthes aspera* on immune organs in arthritic rats.

Treatment	Dose (mg/kg)	Thymus index	Spleen index
Control	—	0.44 ± 0.008	2.88 ± 0.046

FCA control	—	0.62 ± 0.017^#^	3.80 ± 0.055^#^

Indomethacin	1	0.48 ± 0.011^*∗∗∗*^	3.10 ± 0.089^*∗∗∗*^

SR fraction	50	0.56 ± 0.014^*∗*^	3.30 ± 0.083^*∗∗∗*^
	100	0.52 ± 0.009^*∗∗∗*^	3.14 ± 0.056^*∗∗∗*^

Values are presented as mean ± SEM, *n* = 6.

^*∗*^ *P* < 0.05 and ^*∗∗∗*^ *P* < 0.001 compared with the FCA control group.

^#^ *P* < 0.05 compared with vehicle control.

**Table 3:** Effect of saponin rich (SR) fraction of *Achyranthes aspera* on total and differential leucocyte count of arthritic rats.

Treatment	Dose (mg/kg)	Haematological parameters ± SEM					
		Total count (×10^3^/cmm)	Neutrophils (%)	Eosinophils (%)	Lymphocytes (%)	Monocytes (%)	Platelets (×10^5^/*μ*L)
Control	—	9.03 ± 0.625	22.17 ± 2.136	2.33 ± 0.333	75.00 ± 1.983	0.50 ± 0.224	8.98 ± 0.499

FCA control	—	10.65 ± 0.650	26.83 ± 2.522	3.17 ± 0.601	69.67 ± 2.578	0.33 ± 0.211	7.92 ± 0.317

Indomethacin	1	7.92 ± 0.529^*∗∗*^	21.00 ± 1.155	2.67 ± 0.333	75.67 ± 1.116	0.67 ± 0.211	8.27 ± 0.438

SR fraction	50	9.38 ± 0.476	23.83 ± 1.641	3.00 ± 0.365	72.83 ± 1.621	0.33 ± 0.211	9.10 ± 0.593
	100	8.92 ± 0.456	21.83 ± 2.023	2.83 ± 0.601	74.83 ± 2.442	0.50 ± 0.342	8.98 ± 0.464

Values are presented as mean ± SEM, *n* = 6.

^*∗∗*^ *P* < 0.01 compared with the FCA control group.

**Table 4:** Effect of saponin rich (SR) fraction of *Achyranthes aspera* on indexes of oxidative stress in liver of arthritic rats.

Treatment	Dose (mg/kg)	SOD (U/mL/mg of protein)	CAT (U/mL/mg of protein)	GSH (*μ*g/mL/mg of protein)	MDA (nMol/mL/mg of protein)	Nitric oxide (*μ*Mol/mL/mg of protein)
Control	—	20.44 ± 0.64	179.62 ± 5.42	21.96 ± 1.32	7.06 ± 0.514	11.78 ± 0.91

FCA Control	—	4.72 ± 0.60^#^	88.83 ± 1.83^#^	6.99 ± 0.76^#^	16.61 ± 0.742^#^	90.72 ± 2.86^#^

Indo	1	10.34 ± 0.50^*∗∗∗*^	122.98 ± 3.51^*∗*^	12.83 ± 0.82^*∗∗*^	10.64 ± 0.102^*∗∗∗*^	32.22 ± 1.66^*∗∗∗*^

SR fraction	50	13.37 ± 0.91^*∗∗∗*^	127.85 ± 2.80^*∗∗∗*^	15.65 ± 0.95^*∗∗∗*^	13.71 ± 0.858	40.19 ± 1.79^*∗∗∗*^
	100	16.75 ± 1.41^*∗∗∗*^	139.08 ± 2.54^*∗∗∗*^	15.92 ± 1.21^*∗∗∗*^	9.22 ± 0.735^*∗∗∗*^	33.81 ± 2.21^*∗∗∗*^

Values are presented as mean ± SEM, *n* = 6.

^*∗*^ *P* < 0.05, ^*∗∗*^ *P* < 0.01, and ^*∗∗∗*^ *P* < 0.001 compared with the FCA control group.

^#^ *P* < 0.05 compared with vehicle control.

**Table 5:** Effect of saponin rich (SR) fraction of *Achyranthes aspera* on levels of tissue marker enzymes in arthritic rats serum.

Treatment	Dose (mg/kg)	AST (U/L)	ALT (U/L)	ALP (U/L)
Control	—	152.64 ± 11.48	141.85 ± 9.40	118.47 ± 6.05

FCA control	—	220.01 ± 11.32^#^	217.90 ± 13.86^#^	205.25 ± 6.75^#^

Indo	1	159.22 ± 7.06^*∗∗*^	149.48 ± 6.95^*∗∗∗*^	136.98 ± 8.05^*∗∗∗*^

SR fraction	50	173.43 ± 15.72^*∗*^	178.16 ± 6.26^*∗*^	163.34 ± 10.19^*∗∗*^
	100	164.74 ± 6.68^*∗∗*^	163.16 ± 10.08^*∗∗*^	155.31 ± 9.89^*∗∗*^

Values are presented as mean ± SEM, *n* = 6.

^*∗*^ *P* < 0.05, ^*∗∗*^ *P* < 0.01, and ^*∗∗∗*^ *P* < 0.001 compared with the FCA control group.

^#^ *P* < 0.05 compared with vehicle control.
